# Deconstructing neutrophil to lymphocyte ratio (NLR) in early breast cancer: lack of prognostic utility and biological correlates across tumor subtypes

**DOI:** 10.1007/s10549-024-07286-x

**Published:** 2024-03-07

**Authors:** Esmeralda Garcia-Torralba, Miguel Pérez Ramos, Alejandra Ivars Rubio, Esther Navarro Manzano, Noel Blaya Boluda, Miguel Lloret Gil, Alberto Aller, Pilar de la Morena Barrio, Elisa García Garre, Francisco Martínez Díaz, Francisco García Molina, Asunción Chaves Benito, Elena García-Martínez, Francisco Ayala de la Peña

**Affiliations:** 1 Department of Medical Oncology, Hospital Universitario Morales Meseguer, Murcia, 30008 Spain; 2 Department of Medicine, Medical School, University of Murcia, Murcia, 30001 Spain; 3 Instituto Murciano de Investigación Biosanitaria, IMIB, Murcia, 30120 Spain; 4 Department of Pathology, Hospital Universitario Morales Meseguer, Murcia, 30008 Spain; 5 Centro Regional de Hemodonación, Murcia, 30003 Spain; 6 Department of Pathology, Hospital Universitario Reina Sofía, Murcia, 30003 Spain; 7 Department of Pathology, Medical School, University of Murcia, Murcia, 30001 Spain; 8 Medical School, Universidad Católica San Antonio, Murcia, 30107 Spain; 9 Department of Medical Oncology, School of Medicine, Hospital Universitario Morales Meseguer, University of Murcia, Avda. Marqués de los Vélez, s/n, Murcia, 30008 Spain

**Keywords:** Early breast cancer, Neutrophil-to-lymphocyte ratio, Tumor infiltrating lymphocytes, Prognosis

## Abstract

**Purpose:**

The prognostic utility and biological correlates of neutrophil to lymphocyte ratio (NLR), a potential biomarker of the balance between immune response and the inflammatory status, are still uncertain in breast cancer (BC).

**Methods:**

We analysed a cohort of 959 women with early breast cancer, mostly treated with neoadjuvant or adjuvant chemotherapy. Clinical and pathological data, survival, NLR (continuous and categorical) and stromal tumor infiltrating lymphocytes (sTIL) were evaluated.

**Results:**

NLR was only weakly associated with Ki67, while no association was found for grade, histology, immunohistochemical subtype or stage. Lymphocyte infiltration of the tumor did not correlate with NLR (Rho: 0.05, *p* = 0.30). These results were similar in the whole group and across the different BC subtypes, with no differences in triple negative BC. Relapse free interval (RFI), breast cancer specific survival (BCSS) and overall survival (OS) changed according to pre-treatment NLR neither in the univariate nor in the multivariate Cox models (RFI: HR 0.948, *p* = 0.61; BCSS: HR 0.920, *p* = 0.57; OS: HR 0.96, *p* = 0.59).

**Conclusion:**

These results question the utility of NLR as a prognostic biomarker in early breast cancer and suggest the lack of correlation of NLR with tumor microenvironment immune response.

**Supplementary Information:**

The online version contains supplementary material available at 10.1007/s10549-024-07286-x.

## Introduction

The recent understanding of tumor immunosurveillance as a limitation for tumor development and growth and the introduction of immunotherapy has lead to the search of intermediate immune biomarkers of cancer recurrence and progression. Although the tumor immune microenvironment plays a key role in the response to different cancer treatments and in prognosis [1], there is growing evidence that the integrity of the peripheral immune system is also mandatory for an effective immunosurveillance [2]. Previous researchers have proposed the neutrophil-to-lymphocyte ratio (NLR) as an easily available predictor of adverse clinical outcome in several solid tumors [3] and in other diseases in which the systemic inflammation and physiological stress may play a pathogenic role [4, 5]. This marker is particularly interesting since it ideally integrates the subject’s immune response capacity with both the inflammatory status and the neutrophil presence, which tend to correlate with tumour progression and poor prognosis [6, 7]. Moreover, it might reflect the patient’s systemic or peripheral immune status, which is probably interrelated with, but not equivalent to that observed in the tumour microenvironment [2].

Breast cancer (BC) is the leading cause of cancer death in women [8]. Despite most patients are diagnosed with early BC, up to 30% of these cases will relapse in the first 10 years of follow-up. Since current prognostic models for relapse and survival are imperfect, leading to adjuvant or neoadjuvant overtreatment of many patients [9], the identification of other prognostic factors is necessary to safely escalate or descalate adjuvant and neoadjuvant treatment [10]. The role of the immune response in BC has not been fully elucidated, but the recent introduction of immunotherapy both in early and advanced BC [11] together with a better understanding of the contribution of immune microenvironment to chemotherapy and targeted treatment response has fostered the search for new immune response-related biomarkers. Among them, the NLR has been explored as a biomarker of prognosis and response in BC, especially in the early BC setting, in which an elevated NLR has been associated with decreased survival [12]. However, despite the meta-analysis data showing its prognostic significance, no validated early BC prognostic model has incorporated it [13], nor has it been recognized as a useful biomarker for clinical use, not even in the most immunogenic triple negative BC subtype [14]. Furthermore, its significance in metastastic BC is unclear [15].

There also exists significant uncertainty regarding the biological meaning of the NLR in early BC, in which leukocytosis could be associated with other patient-related factors [16] and some studies have demonstrated the prognostic impact of lymphopenia both before and after treatment [17]. This uncertainty is increased by the fact that the NLR values reported as significant frequently overlaps with those found in the normal population [18], and they clearly fall below the usual cut-points for unfavorable prognosis in other tumors [19, 20]. Furthermore, the association of NLR with other immune biomarkers, particularly tumor infiltrating lymphocytes (TIL) [21], remains underexplored. The available studies, usually with a small sample size and significant heterogeneity, have not established any link between NLR and the immune response in the primary tumor [22, 23].

Thus, the aim of this original research was to determine the clinical meaning and the biological correlates of NLR as a peripheral immune biomarker, with the biological characteristics, especially the lymphocytic infiltration, of the primary tumor across subtypes of early BC.

## Methods

### Study design

An observational single-center prospective cohort of 959 women with early breast cancer (2012–2020) was retrospectively analyzed. Inclusion criteria for this study were female sex, histologic diagnosis of early breast cancer (stages I-III), availability of pre-treatment blood cell count and signed informed consent for the study. For the biomarker sub-study, availability of pre-treatment core biopsy for sTIL evaluation was also required. Written informed consent was obtained from all patients included in the study. The study was approved by the Clinical Research and Trials Committee of the University Hospital Morales Meseguer (Internal code: EST08/21) and was conducted in accordance with the ethical principles of the Declaration of Helsinki. The primary endpoints were NLR, sTIL percentage in core biopsy, relapse free interval, and breast cancer-specific survival, as defined below. Overall survival was a secondary endpoint.

### Cohort assembly, diagnosis, and treatment

Patients were obtained from a previous prospective cohort of 1006 consecutive breast cancer included, between 2012 and 2018 in a translational study in which most patients received adjuvant or neoadjuvant chemotherapy. Of the cohort of 1006 patients, 6 male patients and 41 patients without pre-treatment NLR data were excluded (Supplementary Fig. 1), with a final sample size of 959. Diagnosis and treatment were performed according to standard clinical practice [24]. Chemotherapy schedules were classified as 2nd generation (doxorubicin-cyclophosphamide or docetaxel-cyclophosphamide) or 3rd generation (sequential or concurrent anthracyclines and taxanes) regimens.

### Neutrophil-to-lymphocyte ratio calculation

Routine laboratory parameters were collected from laboratory databases, using the closest blood count prior to the date of surgery in the adjuvant cohort or to the date of neoadjuvant chemotherapy (NCT) initiation (maximum time: two weeks). Pretreatment NLR was calculated by dividing the absolute neutrophil count by the absolute lymphocyte count.

### sTIL analysis and pathologic evaluation

Pre-treatment sTIL were measured by a single expert breast pathologist (MPR) blinded to the patients’ outcomes. sTIL was expressed as the percentage of TIL in the intratumoral stromal area of H&E stained slides from the diagnostic core biopsy. The whole stroma compartment within the borders of the invasive breast tumor (excluding necrotic or artifacted zones) was considered for sTIL quantification. The sTIL quantitative variable correspond to the percentage of the area occupied by lymphocytes related to the total area of stromal tissue. Published standard methods from the International sTIL Working Group were followed for sTIL evaluation [25]. sTIL values were either expressed as a continuous variable (percentage) or categorized in three groups (sTIL < 1%, 1–10%, > 10%). Evaluation of immunohistochemistry for estrogen receptors (ER), progesterone receptors (PgR), HER2 and Ki-67 was performed following standard validated procedures [26, 27]. Ki67 was scored globally (average count) and expressed as a continuous variable, withoud categorization. Tumors were classified according to immunohistochemical surrogate molecular subtypes: triple negative breast cancer (ER negative, PgR negative, HER2 negative), HER2+/HR- (HER2 positive, ER negative, PgR negative), HER2+/HR+ (HER2 positive, ER and/or PgR positive), and HR+/HER2- (luminal) tumors. HR + HER2- tumors were further classified as either luminal B tumors (high Ki-67 level, defined as Ki-67 > 14%, or grade 3 or negative PgR, defined as < 20%) or as luminal A tumors (Ki-67 ≤ 14% and grade 1–2 and positive PgR).

In those patients treated with NCT, pathological complete response (pCR) was defined as the absence of invasive carcinoma in the breast and axilla, regardless of the presence of carcinoma in situ (ypT0/Tis ypN0).

### Sample size and power estimation

Assuming a censoring rate of 90% and a maximum two-sided alpha error of 0.05, the study (*n* = 959) had 80% power to detect a hazard ratio (HR) of at least 2.0 for overall survival between two groups (1:1) defined by dichotomic NLR (cut point: median NLR). Thus, the study had an adequate power to detect moderate differences in OS, according to prior reported HR for OS for NLR in early BC [12]. An additional analysis of dichotomic NLR using a pre-defined cut-off of 3 (in agreement with data from previous meta-analysis) was planned.

### Statistical analysis

We followed the REMARK guidelines for the analysis and report of our results [28]. Descriptive analyses of qualitative variables included proportions. Shapiro-Wilk tests were used to test continuous variables for normality. Continuous variables with normal distribution were presented as means ± standard deviations (SD), whereas non-normally distributed variables were reported as median and interquartile ranges (IQR). Pearson’s χ2 test was used to compare proportions or ordinal variables. Differences in means were studied with the Student’s t-test (parametric) or the Mann-Whitney U test (non-parametric). The correlation between NLR and sTIL overall and by subtypes were evaluated with non-parametric test (Spearman’s Rho); values of Rho < 0.200 were not considered relevant.

The main outcome variables were relapse free interval (RFI), defined, according to STEEP criteria [29], as the interval between the date of the first treatment (either surgery or first cycle of neoadjuvant chemotherapy) and the date of distant or locoregional invasive relapse or death by breast cancer, and breast cancer-specific survival (BCSS), calculated from the date of the first treatment to the date of death by breast cancer. Overall survival (OS) was also calculated from the date of the first treatment to the date of death by any cause. Median follow-up was calculated with the inverse Kaplan-Meier method. For survival analyses of RFI, BCSS and OS we used the Kaplan-Meier method and log-rank tests. The prognostic impact of the different clinical and biological variables on the outcome was ascertained by uni- and multivariate Cox proportional hazards regression models. The selection of variables for the multivariate models was based on theoretical considerations and previous results from the literature. Treatment-related variables were included in the model to obtain valid predictions under non-treatment conditions. To overcome the possibility of collinearity, variables with strong (|r| ≥ 0.5) or significant (*p* < 0.05) correlations were excluded. Biological variables were analysed as continuous quantitative variables. We used the likelihood ratio test (LRT) for comparison of the prognostic performance of nested prognostic models with or without inclusion of NLR as a variable.

All p-values were bilateral. A p-value of < 0.05 was considered statistically significant. Holms-Bonferroni adjustment was used for multiple comparisons. All analyses were performed with R version 4.2.3 and RStudio (version 2023.03.0).

## Results

### Patient characteristics and treatment

A total sample of 959 women with early BC was included in the study (Supplementary Fig. S1). Main patients’ characteristics are shown in Table 1. Median age was 52 years. Approximately half of patients were treated with neoadjuvant chemotherapy (47.7%) and the rest of them (52.3%) underwent primary surgical treatment followed by systemic adjuvant therapy, mostly chemotherapy (67.3%). After a median follow-up of 75 months, 87 deaths (47 due to BC) and 91 relapses (81 distant) occurred. Median breast cancer-specific survival (BCSS) was not reached; 5-year and 10-year BCSS was 96.0% (95%CI: 94.7–97.3%) and 92.9% (95%CI: 90.4–95.4%), respectively. Five-year and 10-year relapse-free interval (RFI) was 92.2% (95%CI: 90.4–93.9%) and 87.9% (95%CI:84.9–90.3%).

**Table 1:** Baseline patient and tumor characteristics

	Total cohort (*n* = 959)
**Age**, median (Q1, Q3), years	52.0 (44.8, 62.9)
**Menopausal status**	
Premenopausal	499 (52.0%)
Postmenopausal	459 (47.9%)
NA	1 (0.1%)
**Histology**	
Invasive ductal carcinoma	864 (90.1%)
Invasive lobular carcinoma	64 (6.7%)
Other subtypes	31 (3.2%)
**Immunohistochemical subtype**	
HR+/HER2-	624 (65.1%)
HR+/HER2+	160 (16.7%)
HR-/HER2+	60 (6.3%)
TNBC	115 (12.0%)
**Ki67**, median (Q1, Q3) percentage	30.0 (15.0, 50.0)
**Grade**	
Grade 1	129 (13.5%)
Grade 2	430 (44.8%)
Grade 3	356 (37.1%)
Unknown	44 (4.6%)
**Tumor stage**	
T1	305 (31.8%)
T2-4	648 (67.6%)
Unknown	6 (0.6%)
**Tumor size**, median (Q1, Q3), mm	27.0 (18.0, 40.0)
**Clinical nodal stage**	
cN0	546 (56.9%)
cN1	217 (22.6%)
cN2-3	188 (19.6%)
NA	8 (0.8%)
**Pathological nodal stage**	
pN0/ypN0	547 (57.0%)
pN+/ypN+	367 (38.3%)
NA	45 (4.7%)
**Treatment setting**	
Adjuvant	502 (52.3%)
Neoadjuvant	457 (47.7%)
**Chemotherapy schedule**	
No chemotherapy	164 (17.1%)
2nd generation CT	260 (27.1%)
3rd generation CT	535 (55.8%)
**Other adjuvant systemic treatment**	
Adjuvant endocrine therapy	770 (80.3%)
Adjuvant trastuzumab therapy	220 (23.0%)

CT: Chemotherapy. HR: Hormone Receptor. TNBC: Triple-Negative Breast Cancer. 2nd generation CT: taxane or anthracycline-based regimens (TC: docetaxel-cyclophosphamide; AC: doxorubicin-cyclophosphamide). 3rd generation CT: sequential/concurrent anthracyclines and taxanes (TAC: docetaxel, doxorubicin, cyclophosphamide; weekly Paclitaxel-Doxorubicin/cyclophosphamide). NA: not available

### NLR distribution and association with other clinical and pathological variables

Median baseline NLR value was 1.9 (Q1-Q3 [interquartilic range], 1.5–2.6). We analyzed the association of NLR, both as a continuous and as a dichotomic variable, with other clinical and pathological variables. As shown in Table 2, a higher NLR was observed in younger and premenopausal patients, while no associations of NLR levels were found for most tumor or patient characteristics. In particular, no differences in NLR were found for the BC subtypes (*p* = 0.751) nor between luminal A and B tumors (*p* = 0.857) within the HR + HER2- subtype (Supplementary Fig. S2).

**Table 2:** Association of baseline NLR with other biological and pathologic variables

	Total	NLR		NLR low	NLR high	
Characteristic	N (%)	Median (Q1, Q3)	P value	N (%)	N (%)	P value^*^
**Total**	965 (100%)			493 (%)	466 (%)	--
**Age**, median (Q1, Q3)	52.0 (44.8, 62.9)	---	---	54.0 (45.0, 63.6)	50.4 (44.5, 61.9)	0.149
**Menopausal status**			< 0.001			0.013
Postmenopausal	499 (52.0%)	1.78 (1.40, 2.48)		283 (57.4%)	216 (46.4%)	
Premenopausal	459 (47.9%)	2.0 (1.56, 2.77)		210 (42.6%)	249 (53.4%)	
**Histology**			0.405			0.651
IDC	864 (90.1%)	1.9 (1.46, 2.62)		437 (88.6%)	427 (91.6%)	
ILC	64 (6.7%)	1.74 (1.47, 2.26)		38 (7.7%)	26 (5.6%)	
Other	31 (3.2%)	1.75 (1.51, 2.8)		18 (3.7%)	13 (2.8%)	
**Subtype**			0.751			0.936
HR+/HER2-	624 (65.1%)	1.89 (1.47, 2.59)		320 (64.9%)	304 (65.2%)	
HR+/HER2+	160 (16.7%)	1.91 (1.5, 2.59)		80 (16.2%)	80 (17.2%)	
HR-/HER2+	60 (6.3%)	1.89 (1.58, 2.95)		31 (6.3%)	29 (6.2%)	
TNBC	115 (12.0%)	1.85 (1.42, 2.59)		62 (12.6%)	53 (11.4%)	
**ER**			0.841			0.695
Negative	190 (19.8%)	1.86 (1.43, 2.62)		101 (20.5%)	89 (19.1%)	
Positive	768 (80.1%)	1.89 (1.49, 2.6)		391 (79.3%)	377 (80.9%)	
**PgR**			0.600			0.651
Negative	306 (31.9%)	1.85 (1.47, 2.57)		166 (33.7%)	140 (30.0%)	
Positive	652 (68.0%)	1.91 (1.47, 2.62)		326 (66.1%)	326 (70.0%)	
**HER2**			0.413			0.791
Negative	739 (77.1%)	1.88 (1.45, 2.59)		382 (77.5%)	357 (76.6%)	
Positive	220 (22.9%)	1.9 (1.54, 2.69)		111 (22.5%)	109 (23.4%)	
**Ki67**, median (Q1, Q3)	30.0 (15.0, 50.0)		---	25.0 (10.8, 50.0)	30.0 (18.5, 50.0)	0.049
**Grade**			0.207			0.643
Grade 1	129 (13.5%)	1.84 (1.44, 2.67)		69 (14.0%)	60 (12.9%)	
Grade 2	430 (44.8%)	1.83 (1.43, 2.52)		233 (47.3%)	197 (42.3%)	
Grade 3	356 (37.1%)	1.95 (1.5, 2.62)		170 (34.5%)	186 (39.9%)	
Missing	44 (4.6%)			21 (4.3%)	23 (4.9%)	
**Tumor size (mm)**, median (Q1, Q3)	27.0 (18.0, 40.0)	---	---	27.0 (18.0, 42.0)	27.0 (19.0, 39.0)	0.910
Missing	6 (0.6%)			4 (0.8%)	2 (0.4%)	
**cN**			0.370			0.639
cN0	546 (56.9%)	1.87 (1.46, 2.59)		283 (57.4%)	263 (56.4%)	
cN1	217 (22.6%)	1.95 (1.5, 2.79)		102 (20.7%)	115 (24.7%)	
cN2-3	188 (19.6%)	1.83 (1.44, 2.45)		103 (20.9%)	85 (18.2%)	
Missing	8 (0.8%)			5 (1.0%)	3 (0.6%)	
**pN/ypN**			0.514			0.789
pN0/ypN0	550 (57.5%)	1.9 (1.48, 2.63)		277 (56.2%)	273 (58.6%)	
pN+/ypN+	376 (39.3%)	1.88 (1.44, 2.57)		199 (40.4%)	177 (38.0%)	
pNx/ypNx	30 (3.1%)	1.89 (1.60, 2.63)		17 (3.4%)	16 (3.4%)	

NOTE: *Adjusted p (Bonferroni-Holms)

CT: Chemotherapy. ER: Estrogen Receptor. HR: Hormone Receptor. IDC: Invasive Ductal Carcinoma. ILC: Invasive Lobular Carcinoma. PgR: Progesterone Receptor. TNBC: Triple-negative Breast Cancer

NLR did not correlate with estrogen receptor (Rho, 0.010; *p* = 0.80) or progesterone receptor (Rho, 0.05; *p* = 0.10) percentage, but proliferation, as determined by Ki67%, was weakly associated with NLR (Spearman’s Rho, 0.102; *p* = 0.002) (Supplementary Fig. S3). Ki67 was also higher (*p* = 0.049) in patients with high NLR (median Ki67, 30%; Q1-Q3: 18–50%) than in patients with low NLR (median Ki67, 25%; Q1-Q3: 11–50%).

### Correlation of NLR and stromal tumor infiltrating lymphocytes (sTIL) across breast cancer molecular subtypes

We sought to determine the correlation between NLR, as a peripheral blood immune biomarker, and sTIL, as the most usual immune biomarker in the primary tumor. The analysis of 535 patients, in which both variables were available (Supplementary Table 1), showed a median tumor stromal lymphocytic infiltration of 5% (Q1-Q3: 4–15), with significant differences between immunohistochemical surrogate BC molecular subtypes (*p* < 0.001): higher values of sTIL were found in triple negative breast cancer (median 10%, Q1-Q3: 5–36) and in HR-/HER2 + tumors (median 20%, Q1-Q3: 5–30), while luminal BC cases showed lower infiltration (median 5%, Q1-Q3: 0–10).

We did not find a significant correlation between NLR and sTIL neither in the whole cohort (Rho, 0.05; *p* = 0.30) (Supplementary Fig. S3) nor in the different BC subtypes (Table 3).

**Table 3:** NLR and sTIL global and by subtypes

	Total	sTIL	NLR		NLR low	NLR high	
Characteristic	N (%)	Median sTIL (Q1, Q3)	Median RNL (Q1, Q3)	P value	N (%)	N (%)	P value^*^
**Total**	535 (100%)				257 (%)	278 (%)	
		5 (4, 15)	1.95 (1.5, 2.7)	---	5 (5, 15)	10 (3, 15)	0.776
**sTIL group**				0.806			0.820
sTIL < 1%	125 (23.4%)	---	1.95 (1.45, 2.52)		60 (48%)	65 (52%)	
sTIL 1–10%	260 (48.6%)	---	1.93 (1.5, 2.78)		128 (49.2%)	132 (50.8%)	
sTIL > 10%	150 (28.0%)	---	2 (1.58, 2.71)		69 (46%)	81 (54%)	
**sTIL group by subtype**				0.256			0.381
**HR+/HER2-**	335 (62.6%)	5 (0, 10)	1.95 (1.48, 2.64)	0.698	162 (48.4%)	173 (51.6%)	0.507
sTIL < 1%			1.96 (1.44, 2.52)		48 (47.5%)	53 (52.5%)	
sTIL 1–10%			1.89 (1.48, 2.75)		90 (50.8%)	87 (49.2%)	
sTIL > 10%			2.07 (1.59, 2.76)		24 (42.1%)	33 (57.9%)	
**HR+/HER2+**	90 (16.8%)	10 (5, 20)	2.06 (1.63, 2.84)	0.895	38 (42.2%)	52 (57.8%)	0.593
sTIL < 1%			2.11 (1.86, 2.49)		2 (25%)	6 (75%)	
sTIL 1–10%			2.06 (1.63, 2.89)		17 (42.5%)	23 (57.5%)	
sTIL > 10%			1.95 (1.61, 2.57)		19 (45.2%)	23 (54.8%)	
**HR-/HER2+**	46 (8.6%)	20 (5, 30)	2.12 (1.70, 3.07)	0.272	21 (45.7%)	25 (54.3%)	0.474
sTIL < 1%			1.60 (1.33, 1.93)		3 (75%)	1 (25%)	
sTIL 1–10%			2.11 (1.67, 3.08)		8 (47.1%)	9 (52.9%)	
sTIL > 10%			2.17 (1.79, 3.24)		10 (40%)	15 (60%)	
**TNBC**	64 (12.0%)	10 (5, 36)	1.84 (1.46, 2.57)	0.253	36 (56.2%)	28 (43.8%)	0.694
sTIL < 1%			1.86 (1.52, 2.57)		7 (58.3%)	5 (41.7%)	
sTIL 1–10%			1.95 (1.55, 2.73)		13 (50%)	13 (50%)	
sTIL > 10%			1.69 (1.27, 2.41)		16 (61.5%)	10 (38.5%)	

*Bonferroni-Holms adjustment

HR: Hormone Receptor. NLR: Neutrophil-to-Lymphocyte Ratio. sTIL: stromal Tumor Infiltrating Lymphocytes. TNBC: Triple-negative Breast Cancer

**Fig. 1:**
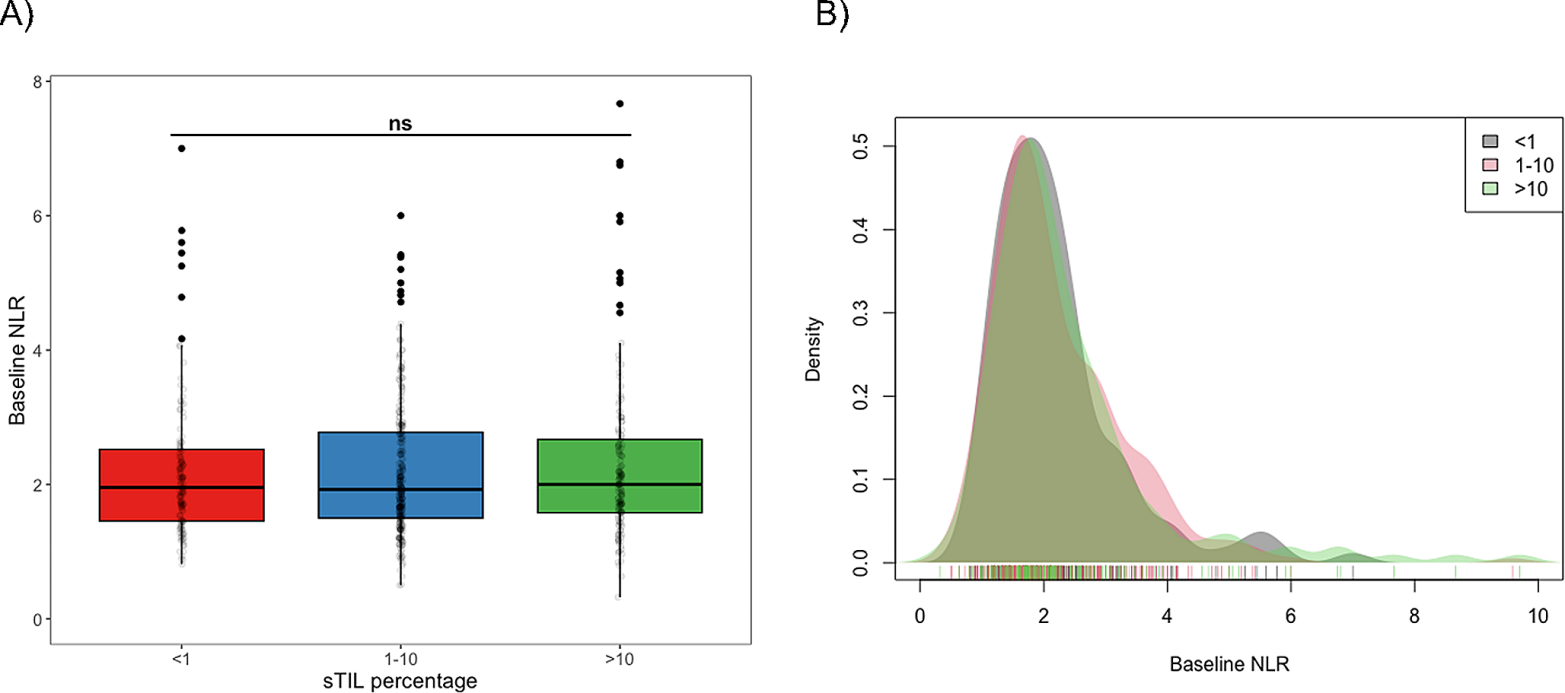
**Values of baseline NLR according to categorical TIL groups in early BC patients** (A) Baseline NLR for the three groups of sTIL (< 1%, 1–10%, > 10%). The central line in each boxplot correspond to the median value of NLR; black dots correspond to outliers; error bars represent ± 1.5 IQR; ns: non-significant p-value (Kruskal-Wallis test). (B) Density histogram for NLR in the three sTIL subgroups

Similarly, the analysis of NLR according to sTIL subgroups did not show any association between both variables (Fig. 1). Categorical analysis of the distribution of NLR high and low groups across sTIL subgroups (Table 3) did not reveal any association (Chi-squared; *p* = 0.820). Similar results were obtained when the analysis was performed in each BC subtype (Table 3).

Since both NLR and sTIL showed a significant correlation with Ki67, we also assessed the concurrent effect of sTIL and Ki67 on NLR. A clear pattern of variability was not identifiable, neither in the whole cohort nor across the four BC subtypes (Supplementary Fig. S4).

### Association of NLR with survival outcomes

The baseline NLR level, considered as a continuous variable, did not show any association with RFI (HR 0.99; 95%CI, 0.84–1.19) or BCSS (HR 0.97; 95%CI, 0.76–1.25). Likewise, higher NLR (using the median value as cut-off), did not exhibit an association with RFI (HR 0.83; 95%CI, 0.55–1.26) or BCSS (HR 0.82; 95%CI, 0.46–1.45) (Supplementary Fig. S5).

Similarly, the incorporation of NLR as a covariate into multivariate Cox models for both RFI and BCSS was not significantly associated with any of the outcomes (Table 4). In addition, its inclusion did not improve the predictions based on conventional clinical and pathological variables (likelihood ratio test [LRT] comparing models with and without NLR: *p* = 0.598 for RFI, and *p* = 0.553 for BCSS). Since previous reports have shown certain associations between NLR, patient characteristics and non-BC mortality, we also examined its potential effect on OS, without finding any significant association in either the univariate (HR 1.00; 95%CI, 0.84–1.19) or multivariate models (Table 4). No interaction between NLR and Ki67 was found in any of the models. The inclusion of sTIL into the multivariate models in those patients with available data for this biomarker (*n* = 535) did not change these results (data not shown).

**Table 4:** Multivariate Cox models including NLR for RFI and OS.

Model	Beta	HR (95% CI)	p-value
**Relapse free interval**			
NLR (continuous)	-0.053	0.948 (0.773, 1.162)	0.608
Postmenopausal	0.237	1.268 (0.772, 2.082)	0.347
T size (cm)	0.019	1.019 (1.010, 1.030)	< 0.001
Nodal disease	1.041	2.83 (1.611, 4.977)	< 0.001
Grade 3	-0.049	0.951 (0.516, 1.753)	0.873
ER (positive)	-0.827	0.437 (0.225, 0.849)	0.015
HER2 (positive)	0.134	1.144 (0.652, 2.007)	0.638
Ki67 (continuous)	0.006	1.006 (0.994, 1.018)	0.335
Mastectomy	0.526	1.691 (0.996, 2.960)	0.065
Chemotherapy (adjuvant or neoadjuvant)	-0.154	0.857 (0.280, 2.618)	0.786
**Breast cancer specific overall survival**			
NLR (continuous)	-0.083	0.920 (0.687, 1.230)	0.572
Postmenopausal	0.594	1.812 (0.889, 3.691)	0.101
T size (cm)	0.016	1.017 (1.003, 1.029)	0.010
Nodal disease	1.241	3.460 (1.585, 7.549)	0.002
Grade 3	0.255	1.291 (0.562, 2.963)	0.547
ER (positive)	-0.844	0.429 (0.175, 1.054)	0.065
HER2 (positive)	0.109	1.115 (0.509, 2.443)	0.784
Ki67 (continuous)	0.009	1.009 (0.992, 1.026)	0.282
Mastectomy	0.788	2.199 (1.000, 4.836)	0.049
Chemotherapy (adjuvant or neoadjuvant)	-1.043	0.352 (0.089, 1,389)	0.136
**Overall survival**			
NLR (continuous)	-0.045	0.956 (0.812, 1.126)	0.592
Age (continuous)	0.054	1.056 (1.023, 1.090)	< 0.001
Postmenopausal	0.911	2.487 (1.040, 5.948)	0.040
T size (cm)	0.009	1.009 (0.998, 1.021)	0.087
Nodal disease	1.079	2.944 (1.711, 5.066)	< 0.001
Grade 3	0.318	1.374 (0.727, 2.597)	0.327
ER (positive)	-0.574	0.563 (0.280, 1.131)	0.106
HER2 (positive)	0.229	1.257 (0.686, 2.303	0.458
Ki67 (continuous)	0.011	1.011 (0.989, 1.024)	0.078
Mastectomy	0.726	2.067 (1.197, 3.571)	0.009
Chemotherapy (adjuvant or neoadjuvant)	-1.427	0.240 (0.108, 0.531)	< 0.001

CI: Confidence Interval. ER: Estrogen Receptor. HR: Hazard Ratio. NLR: Neutrophil-to-Lymphocyte Ratio

Finally, given that previous studies have indicated a different meaning of NLR according to BC subtype and have used diverse thresholds for dichotomic NLR, we performed additional analyses. A cut-off of 3 for NLR, like that reported in the meta-analysis [12], and corresponding to 85th percentile in our cohort, did not yield differences in either OS (*p* = 0.23), BCSS (*p* = 0.48) or RFI (*p* = 0.60) (Supplementary Fig. S6). Similarly, survival analysis within each BC subtype did not show any association between NLR and patient outcomes, either using dichotomic NLR (Supplementary Fig. S7) or continuous NLR (Supplementary Table 2).

## Discussion

The NLR has been proposed as a prognostic marker in early breast cancer, with higher values indicating a pro-inflammatory and immune-suppressed state [4] associated with a poorer prognosis in terms of relapse and survival [12]. However, the uncertainties surrounding its biological significance and the omission of baseline NLR as a covariate in the validated prognostic models of early breast cancer [13] prompted this study of a cohort of early BC patients treated with contemporary adjuvant and neoadjuvant standards. Beyond the absence of prognostic impact of NLR in early BC patients, the primary finding of this study is the lack of correlation between NLR and tumor lymphocytic infiltration and between NLR and other tumor characteristics.

NLR was only elevated in premenopausal patients, an unexpected finding since higher NLR levels have been reported in older women [30]. We did not identify any association of NLR with higher tumor stage or more aggressive biological traits, except for a weak association with tumor proliferation as assessed through Ki67 immunohistochemistry, the implications of which also remain uncertain. The assessment of the correlation between NLR and sTIL represented the main objective of this study, considering the relevance of understanding the biological correlation of peripheral and tissue immune-related prognostic factors to improve its clinical applicability. In this work, no association between both immune markers was found. The correlation between NLR and sTIL has been previously evaluated, also without finding any link between them. However, the evidence has been limited until now, with studies characterized by small sample size and predominantly focused on TNBC [22, 23, 31–33]. Thus, our work confirms the absence of a relationship between two widely used immune biomarkers, NLR and sTIL, across all subtypes of early BC. These results remained unchanged when variables were analyzed categorically and in analyses stratified by subtypes and levels of lymphocytic infiltration. These findings strongly suggest the lack of association between NLR levels and the degree of immune response activation in the tumor.

The cutoff for NLR is a relevant issue for the interpretation of our data. In this study we decided to analyse NLR as a continuous variable to avoid the loss of information caused by dichotomization and the usual overfitting for models developed using ROC curves to determine the cutoff. However, the analysis with both the median value and 3 as cut points did not show any difference in the risk of death or recurrence. These cutoffs are in fact below the usual cutoff of 4 used in other tumors with higher inflammatory activation, such as pancreatic or renal cancer [19, 20]. It is noteworthy that the median NLR in our cohort was 1.96, close to the median range of 1.60–1.80 which is generally considered as normal in population-based studies [18]. While inflammatory activation contributes to BC progression [34], the observed NLR levels might suggest a comparatively less inflammatory systemic tumor environment in early BC patients. This also suggest a lesser degree of impairment in the anti-tumor immune response compared with other neoplasms or with advanced disease. In this context, we could expect a lower impact of NLR in the prognosis of early BC, except in cases with extreme values.

The patient population included in this study displays the expected distribution of tumor subtypes in early BC. Our data indicate no difference of NLR levels among BC subtypes, nor do they show any different prognostic impact within any of them, including TNBC, for which prior reports have suggested a greater relevance of NLR. These results disagree with those reported by Jia et al. in a Chinese cohort of 1570 patients, mostly treated with adjuvant chemotherapy, and in which a NLR cutoff of 2 was used [35]. However, the referenced study entirely excluded patients treated with neoadjuvant chemotherapy, who represents approximately half of our cohort. Several other studies with sample sizes exceeding 500 patients have similarly shown a prognostic value of NLR in early BC. It is noteworthy, however, that these studies solely considered patients treated in the adjuvant setting. Additionally, certain confusion factors, such as the inclusion of in situ carcinomas [36] or the administration of NSAIDs [37], were present. Another study conducted by Koh et al., the largest reported thus far, also found a relevant prognostic impact for NLR in TNBC [38]. Remarkably, this is the only study that, similarly to ours, includes patients treated in the neoadjuvant setting (although only 15%) and incorporates treatment-related variables in the multivariate model. However, between 16% and 32% of patients within 4th and 5th quintiles of NLR (quintiles showing a difference in terms of DFS and OS) had metastatic disease at time of inclusion. The divergence in NLR’s prognostic impact between these studies and our findings might also be attributed to differences in inclusion periods (all of them before 2011), which correspond to different therapeutic standards, as well as to the lack of inclusion in all of them of some prognostic factors such as Ki67 and grade. Finally, the geographical origin of the patients may have also played a relevant role, considering that a recent meta-analysis, focused on the neoadjuvant setting, showed significant disparities in the prognostic impact of NLR according to geographic location. Interestingly, no effect of NLR on DFS was found in non-Asian populations [39]. In any event, our results do not replicate the previous reported differences in the risk of recurrence or death and suggest the absence of prognostic value for NLR in the context of early BC treated with contemporary adjuvant or neoadjuvant standards when accounting for other prognostic variables in the multivariate models.

Our work has several limitations. First, we examined a high-risk BC cohort, predominantly composed of patients treated with adjuvant or neoadjuvant chemotherapy, although this is precisely the clinical context in which new biomarkers are more necessary to allow new escalation or de-escalation therapeutic strategies. Second, the sample size and number of events gave us adequate statistical power to exclude moderate prognostic effects (HR around 2), but we cannot discard milder effects of NLR on the risk of BC recurrence or death. However, the HR reported in NLR studies for early BC fall within the range of 1.50–2.50 in the publication by Koh et al. [38] and tend to be equal or over 2 in most studies [35, 37] and in the main meta-analysis [12]. Third, although sTIL were evaluated according to international standards, the evaluation was performed by a single pathologist. Fourth, sTIL is an imperfect biomarker of immune response in the tumor microenvironment. Although our data demonstrate no association with the NLR levels, the assessment of other immune biomarkers either in the primary tumor or in plasma samples, might allow a better understanding of the role of NLR, if any, in the immune response status of early BC patients.

In **conclusion**, the results of our study did not establish any correlation between NLR and tumor-infiltrating lymphocytes –a biomarker of tumor immune response—nor with other biological factors relevant for tumor progression. Furthermore, we were unable to replicate, within a contemporary Western cohort of early BC patients, prior findings that supported a prognostic impact of NLR, either in the whole group or in any BC subtype. Taken together, our findings suggest that NLR is neither a substantial prognostic factor nor a valid biomarker for tumor immune response, thus questioning its clinical utility in the context of early BC. Perhaps it is time to consider not including NLR in early breast cancer prognostic studies, especially in non-Asian populations.

### Electronic supplementary material

Below is the link to the electronic supplementary material.

Supplementary Material 1

## Data Availability

The data that support the findings the current study are not publicly available due to Spanish law regulations, but are available from the corresponding author on reasonable request.

## References

[1] Klauschen F, Müller K-R, Binder A (2018). Scoring of tumor-infiltrating lymphocytes: from visual estimation to machine learning. Semin Cancer Biol.

[2] Hiam-Galvez KJ, Allen BM, Spitzer MH (2021). Systemic immunity in cancer. Nat Rev Cancer.

[3] Cupp MA, Cariolou M, Tzoulaki I et al (2020) Neutrophil to lymphocyte ratio and cancer prognosis: an umbrella review of systematic reviews and meta-analyses of observational studies. BMC Med 18. 10.1186/S12916-020-01817-1 10.1186/s12916-020-01817-1 PMC7678319 33213430

[4] Buonacera A, Stancanelli B, Colaci M, Malatino L (2022) Neutrophil to lymphocyte ratio: an emerging marker of the relationships between the immune system and diseases. Int J Mol Sci 23. 10.3390/ijms23073636 10.3390/ijms23073636 PMC8998851 35408994

[5] Dolan RD, McSorley ST, Horgan PG (2017). The role of the systemic inflammatory response in predicting outcomes in patients with advanced inoperable cancer: systematic review and meta-analysis. Crit Rev Oncol Hematol.

[6] McAndrew NP, Bottalico L, Mesaros C et al (2021) Effects of systemic inflammation on relapse in early breast cancer. NPJ Breast Cancer 7. 10.1038/S41523-020-00212-6 10.1038/s41523-020-00212-6 PMC7822844 33483516

[7] Hedrick CC, Malanchi I (2022). Neutrophils in cancer: heterogeneous and multifaceted. Nat Rev Immunol.

[8] Kocarnik JM, Compton K, Dean FE (2022). Cancer Incidence, Mortality, Years of Life Lost, Years lived with disability, and disability-adjusted life years for 29 Cancer groups from 2010 to 2019. JAMA Oncol.

[9] Ragusi MAA, van der Velden BHM, van Maaren MC (2022). Population-based estimates of overtreatment with adjuvant systemic therapy in early breast cancer patients with data from the Netherlands and the USA. Breast Cancer Res Treat.

[10] Gianni C, Palleschi M, Merloni F (2022). Potential impact of Preoperative circulating biomarkers on individual Escalating/de-Escalating strategies in early breast Cancer. Cancers (Basel).

[11] Debien V, De Caluwé A, Wang X (2023). Immunotherapy in breast cancer: an overview of current strategies and perspectives. NPJ Breast Cancer.

[12] Ethier JL, Desautels D, Templeton A et al (2017) Prognostic role of neutrophil-to-lymphocyte ratio in breast cancer: a systematic review and meta-analysis. Breast Cancer Res 19. 10.1186/s13058-016-0794-1 10.1186/s13058-016-0794-1 PMC5217326 28057046

[13] Phung MT, Tin Tin S, Elwood JM (2019). Prognostic models for breast cancer: a systematic review. BMC Cancer.

[14] Andre F, Nofisat I, Allison KH (2022). Biomarkers for adjuvant endocrine and chemotherapy in early-stage breast Cancer: ASCO Guideline Update. J Clin Oncol.

[15] Ivars Rubio A, Yufera JC, de la Morena P et al (2019) Neutrophil-lymphocyte ratio in metastatic breast cancer is not an independent predictor of survival, but depends on other variables. Sci Rep 9. 10.1038/s41598-019-53606-3 10.1038/s41598-019-53606-3 PMC6861311 31740715

[16] Mouchemore KA, Anderson RL, Hamilton JA (2018). Neutrophils, G-CSF and their contribution to breast cancer metastasis. FEBS J.

[17] Vicente Conesa MA, Garcia-Martinez E, Gonzalez Billalabeitia E (2012). Predictive value of peripheral blood lymphocyte count in breast cancer patients treated with primary chemotherapy. Breast.

[18] Fest J, Ruiter R, Ikram MA et al (2018) Reference values for white blood-cell-based inflammatory markers in the Rotterdam Study: a population-based prospective cohort study. Sci Rep 8. 10.1038/s41598-018-28646-w 10.1038/s41598-018-28646-w PMC6043609 30002404

[19] Zhou Y, Wei Q, Fan J (2018). Prognostic role of the neutrophil-to-lymphocyte ratio in pancreatic cancer: a meta-analysis containing 8252 patients. Clin Chim Acta.

[20] Yu Y, Qian L, Cui J (2017). Value of neutrophil-to-lymphocyte ratio for predicting lung cancer prognosis: a meta-analysis of 7,219 patients. Mol Clin Oncol.

[21] El Bairi K, Haynes HR, Blackley E (2021). The tale of TILs in breast cancer: a report from the International Immuno-Oncology Biomarker Working Group. NPJ Breast Cancer.

[22] Dong X, Liu C, Yuan J (2010). Prognostic roles of neutrophil-to-lymphocyte ratio and stromal tumor-infiltrating lymphocytes and their relationship in locally Advanced Triple-negative breast Cancer treated with Neoadjuvant Chemotherapy. Breast Care.

[23] Lee KH, Kim EY, Yun JS et al (2018) The prognostic and predictive value of tumor-infiltrating lymphocytes and hematologic parameters in patients with breast cancer. BMC Cancer 18. 10.1186/s12885-018-4832-5 10.1186/s12885-018-4832-5 PMC6167816 30285668

[24] Ayala de la Peña F, Andrés R, Garcia-Sáenz JA (2019). SEOM clinical guidelines in early stage breast cancer (2018). Clin Transl Oncol.

[25] Salgado R, Denkert C, Demaria S (2015). The evaluation of tumor-infiltrating lymphocytes (TILs) in breast cancer: recommendations by an International TILs Working Group 2014. Ann Oncol.

[26] Wolff AC, Somerfield MR, Dowsett M (2023). Human epidermal growth factor receptor 2 testing in breast Cancer: ASCO–College of American pathologists Guideline Update. J Clin Oncol.

[27] Allison KH, Elizabeth M, Hammond H (2020). Estrogen and progesterone receptor testing in breast Cancer: ASCO/CAP Guideline Update. J Clin Oncol.

[28] McShane LM, Altman DG, Sauerbrei W (2006). REporting recommendations for tumor MARKer prognostic studies (REMARK). Breast Cancer Res Treat.

[29] Tolaney SM, Garrett-Mayer E, White J (2021). Updated standardized definitions for efficacy end points (STEEP) in adjuvant breast Cancer clinical trials: STEEP Version 2.0. J Clin Oncol.

[30] Li J, Chen Q, Luo X (2015). Neutrophil-to-lymphocyte ratio positively correlates to Age in Healthy Population. J Clin Lab Anal.

[31] Lusho S, Durando X, Mouret-Reynier MA et al (2021) Platelet-to-lymphocyte ratio is associated with favorable response to neoadjuvant chemotherapy in triple negative breast cancer: a study on 120 patients. Front Oncol 11:678315. 10.3389/fonc.2021.678315 10.3389/fonc.2021.678315 PMC8331686 34367964

[32] Van Berckelaer C, Van Geyt M, Linders S (2020). A high neutrophil-lymphocyte ratio and platelet-lymphocyte ratio are associated with a worse outcome in inflammatory breast cancer. Breast.

[33] Pang J, Zhou H, Dong X (2021). Relationship between the neutrophil to lymphocyte ratio, stromal tumor-infiltrating lymphocytes, and the prognosis and response to Neoadjuvant Chemotherapy in Triple-negative breast Cancer. Clin Breast Cancer.

[34] Greten FR, Grivennikov SI (2019). Inflammation and Cancer: triggers, mechanisms, and consequences. Immunity.

[35] Jia W, Wu J, Jia H et al (2015) The peripheral blood neutrophil-to-lymphocyte ratio is superior to the lymphocyte-to-monocyte ratio for predicting the long-term survival of triple-negative breast cancer patients. PLoS One 10:e0143061. 10.1371/journal.pone.0143061 10.1371/journal.pone.0143061 PMC4666347 26580962

[36] Yao M, Liu Y, Jin H (2014). Prognostic value of preoperative inflammatory markers in Chinese patients with breast cancer. OncoTargets Ther.

[37] Forget P, Bentin C, Machiels JP et al (2014) Intraoperative use of ketorolac or diclofenac is associated with improved disease-free survival and overall survival in conservative breast cancer surgery. Br J Anaesth 113:i82–i87. 10.1093/bja/aet464 10.1093/bja/aet464 24464611

[38] Koh CH, Bhoo-Pathy N, Ng KL (2015). Utility of pre-treatment neutrophil-lymphocyte ratio and platelet-lymphocyte ratio as prognostic factors in breast cancer. Br J Cancer.

[39] Zhou Q, Dong J, Sun Q (2021). Role of neutrophil-to-lymphocyte ratio as a prognostic biomarker in patients with breast cancer receiving neoadjuvant chemotherapy: a meta-analysis. BMJ Open.

