# Supplementary material for: Deconstructing neutrophil to lymphocyte ratio (NLR) in early breast cancer: lack of prognostic utility and biological correlates across tumor subtypes
Source: Breast Cancer Res Treat. 2024 Mar 7;205(3):475–85. doi: 10.1007/s10549-024-07286-x (PMC11101577; doi:10.1007/s10549-024-07286-x)
Supplement: Supplementary file 1 — Supplementary Material 1 [file 10549_2024_7286_MOESM1_ESM.pdf]

Deconstructing neutrophil to lymphocyte ratio (NLR) in early breast cancer: lack of prognostic utility and biological correlates across tumor subtypes

Authors and affiliations: Esmeralda Garcia-Torralba[a,b,c] (esmeralda.garciat@um.es), Miguel Pérez Ramos[d] (miguel.perez3@carm.es), Alejandra Ivars Rubio[a,b,c] (mariaalejandra.ivars@um.es), Esther Navarro Manzano[a,c,e] (esther.navarro3@um.es), Noel Blaya Boluda[a,b,c] (noel.blaya@um.es), Miguel Lloret (miguellloret12@gmail.com)[b], Alberto Aller (alberto.allerg@um.es)[b], Pilar de la Morena Barrio[a,c] (pdb89e@ad.sms.carm.es), Elisa García Garre (elisa.garcia2@carm.es)[a,c], Francisco Martínez Díaz[c,f,g] (fmdiaz@um.es), Francisco García Molina[f,g] (pacogm@um.es), Asunción Chaves Benito[d,g](mariaa.chaves@carm.es), Elena García-Martínez[a,c,h] (helenagarciam@gmail.com), Francisco Ayala de la Peña[a,b,c] (frayala@um.es).

a. Department of Medical Oncology, Hospital Universitario Morales Meseguer, 30008 Murcia, Spain.
b. Department of Medicine, Medical School, University of Murcia, 30001 Murcia, Spain.
c. Instituto Murciano de Investigación Biosanitaria, IMIB, 30120 Murcia, Spain.
d. Department of Pathology, Hospital Universitario Morales Meseguer, 30008 Murcia, Spain.
e. Centro Regional de Hemodonación, 30003 Murcia, Spain.
f. Department of Pathology, Hospital Universitario Reina Sofía, 30003 Murcia, Spain.
g. Department of Pathology, Medical School, University of Murcia, 30001, Murcia, Spain
h. Medical School, Universidad Católica San Antonio, 30107 Murcia, Spain.

Corresponding author
Francisco Ayala de la Peña (ORCID: 0000-0001-6311-920X)
Department of Medical Oncology. Hospital Universitario Morales Meseguer.
School of Medicine. University of Murcia.
Avda. Marqués de los Vélez, s/n. Murcia 30008, Spain
E-mail: frayala@um.es

SUPPLEMENTARY INFORMATION

SUPPLEMENTARY TABLES

Supplementary Table 1. Characteristics of sTIL /NLR cohort.

	**Cohort with sTIL/NLR (n=535)**
**Age,** median (Q1, Q3)	51.0 [43.9, 61.9]
**Menopausal status**	
Premenopausal	258 (48.2%)
Postmenopausal	276 (51.6%)
**Histology**	
Invasive ductal carcinoma	499 (93.3%)
Invasive lobular carcinoma	31 (5.8%)
Other subtypes	5 (0.9%)
**Immunohistochemical subtype**	
HR+/HER2-	335 (62.6%)
HR+/HER2+	90 (16.8%)
HR-/HER2+	46 (8.6%)
TNBC	64 (12.0%)
**Ki67**, median (Q1, Q3) percentage	35.0 [20.0, 50.0]
**Grade**	
Grade 1	49 (9.2%)
Grade 2	242 (45.2%)
Grade 3	210 (39.3%)
**Tumor stage**	
T1	105 (19.6%)
T2-4	425 (79.4%)
**Tumor size**, median (Q1, Q3) mm	31.8 [22.0, 50.0]
**Clinical nodal stage**	
cN0	233 (43.6%)
cN1	143 (26.7%)
cN2	152 (28.4%)
**Pathological nodal stage**	
pN0/ypN0	283 (52.9%)
pN+/ypN+	222 (41.5%)
pNx/ypNx	27 (5.0%)
**Chemotherapy**	
No chemotherapy	56 (10.5%)
2nd generation CT	153 (28.6%)
**3**rd generation CT	326 (60.9%)
**Treatment setting**	
Adjuvant	157 (29.3%)
Neoadjuvant	378 (70.7%)

CT: Chemotherapy. HR: Hormone Receptor. TNBC: Triple-Negative Breast Cancer. 2nd generation CT: taxane or anthracycline-based regimens (TC: docetaxel-cyclophosphamide; AC: doxorubicin-cyclophosphamide). 3rd generation CT: sequential/concurrent anthracyclines and taxanes (TAC: docetaxel, doxorubicin, cyclophosphamide; weekly paclitaxel-doxorubicin/cyclophosphamide).

Supplementary Table 2. Univariate Cox-models for association of NLR (continuous variable) to early breast cancer survival by subtypes.

	**Beta**	**HR (95% CI)**	**p-value**
**Overall survival**			
TNBC	0.034	1.035 (0.636, 1.685)	0.890
HER2+	-0.165	0.848 (0.562, 1.278)	0.431
HR+/HER2-	0.061	1.063 (0.874, 1.293)	0.539
**Breast cancer specific survival**			
TNBC	0.044	1.045 (0.546, 2.001)	0.894
HER2+	-0.245	0.783 (0.440, 1.393)	0.405
HR+/HER2-	0.044	1.045 (0.789, 1.383)	0.758
**Relapse free interval**			
TNBC	-0.008	0.992 (0.567, 1.734)	0.976
HER2+	-0.095	0.909 (0.635, 1.301)	0.603
HR+/HER2-	0.0311	1.032 (0.839, 1.268)	0.768

TNBC: Triple Negative Breast Cancer; HR: Hormone Receptor; HR: Hazard Ratio

SUPPLEMENTARY FIGURES

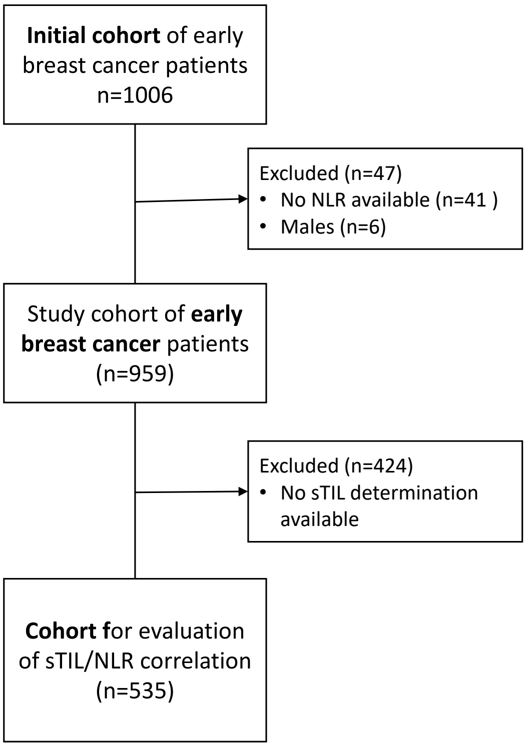

Fig. S1 Consort diagram of the study

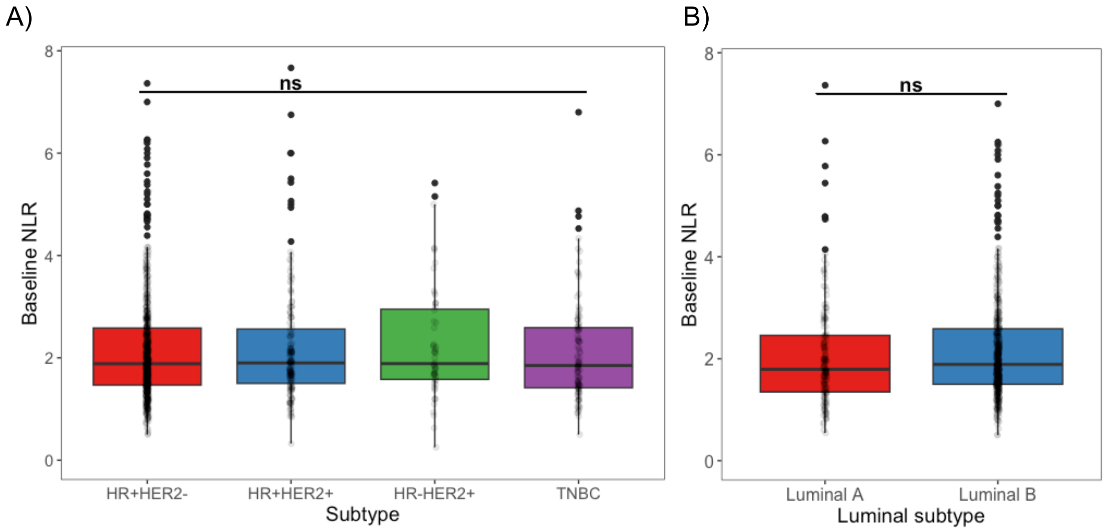

Fig. S2 NLR values in BC subtypes (A) NLR values in the four BC subtypes. (B) NLR values comparison in luminal A and B subtypes of luminal (HR+/HER2-) BC. The central line in each boxplot correspond to the median value of NLR; black dots correspond to outliers; error bars represent ± 1.5 IQR; ns: non-significant p-value (Kruskal-Wallis test).

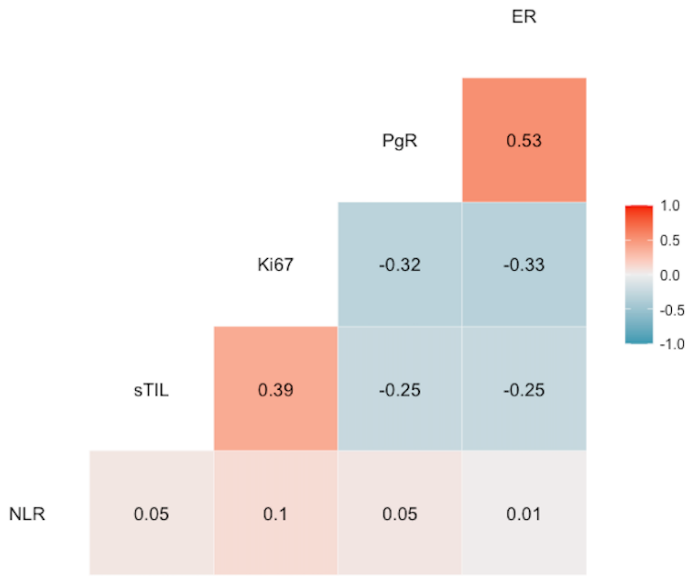

Fig. S3 Correlogram showing the association of NLR with other tumor characteristics Spearman correlation coefficients are showed for each pair of variables. sTIL: stromal Tumor Infiltrating Lymphocytes; PgR: Progesterone Receptor; ER: Estrogen Receptor.

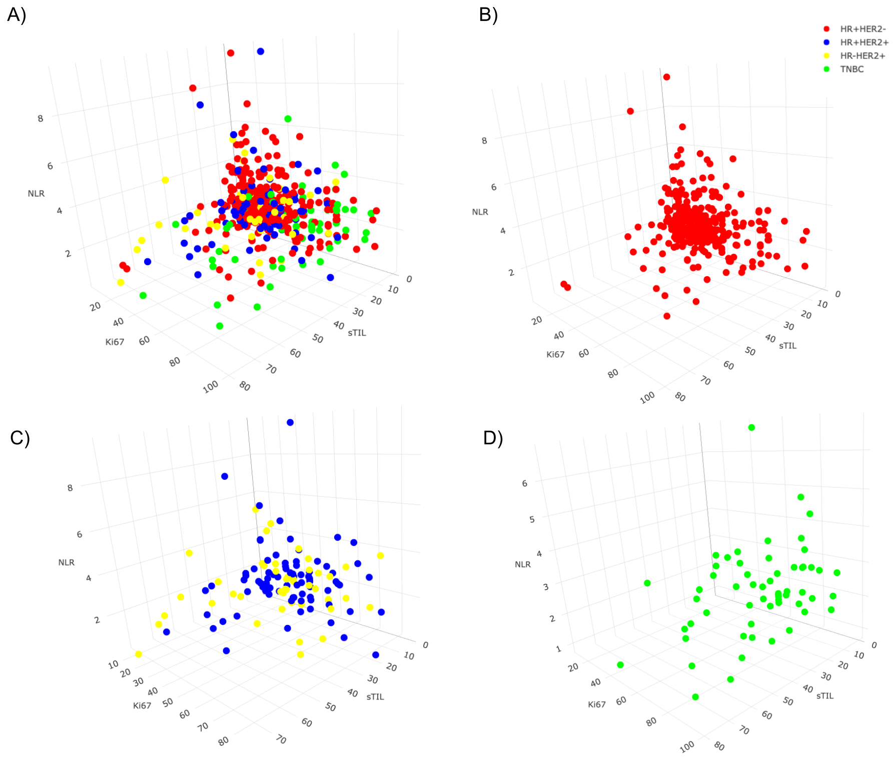

Fig. S4 Distribution of NLR, Ki67 and TIL across BC subtypes (A) Whole cohort. (B) HR+HER2- patients. (C) HER2+ patients. (D) Triple negative patients.

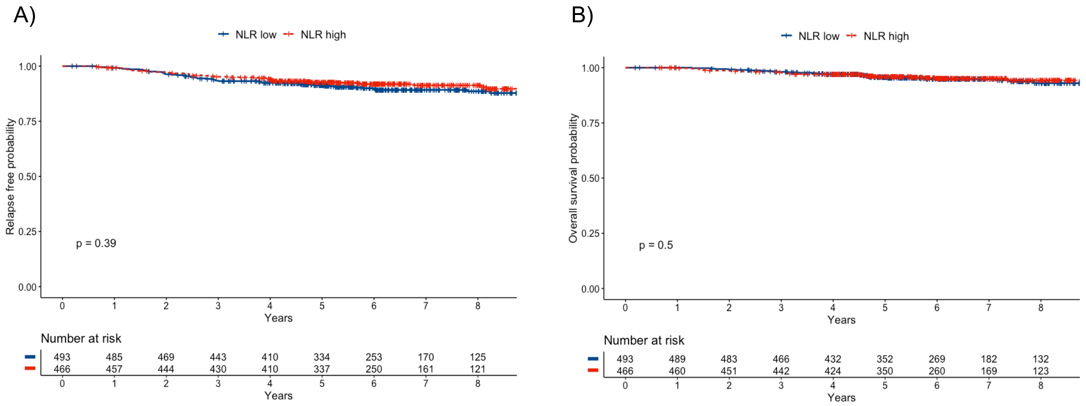

Fig. S5 Kaplan-Meier survival curves according to categorical RNL in early BC patients (A) Relapse free interval. (B) Breast cancer specific survival. P-values correspond to log-rank test.

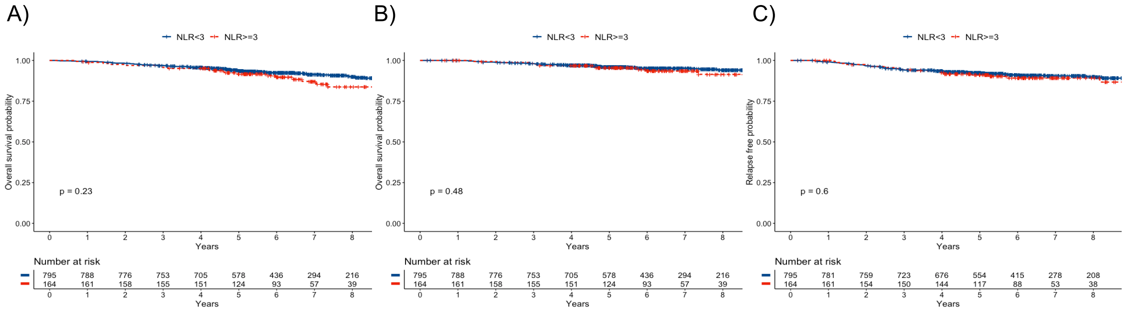

Fig. S6 Kaplan-Meier survival curves for baseline NLR using a cut-off of 3 (A) Overall survival (OS); (B) Breast cancer specific survival (BCSS); (C) Relapse free interval (RFI). P-values correspond to log-rank test.

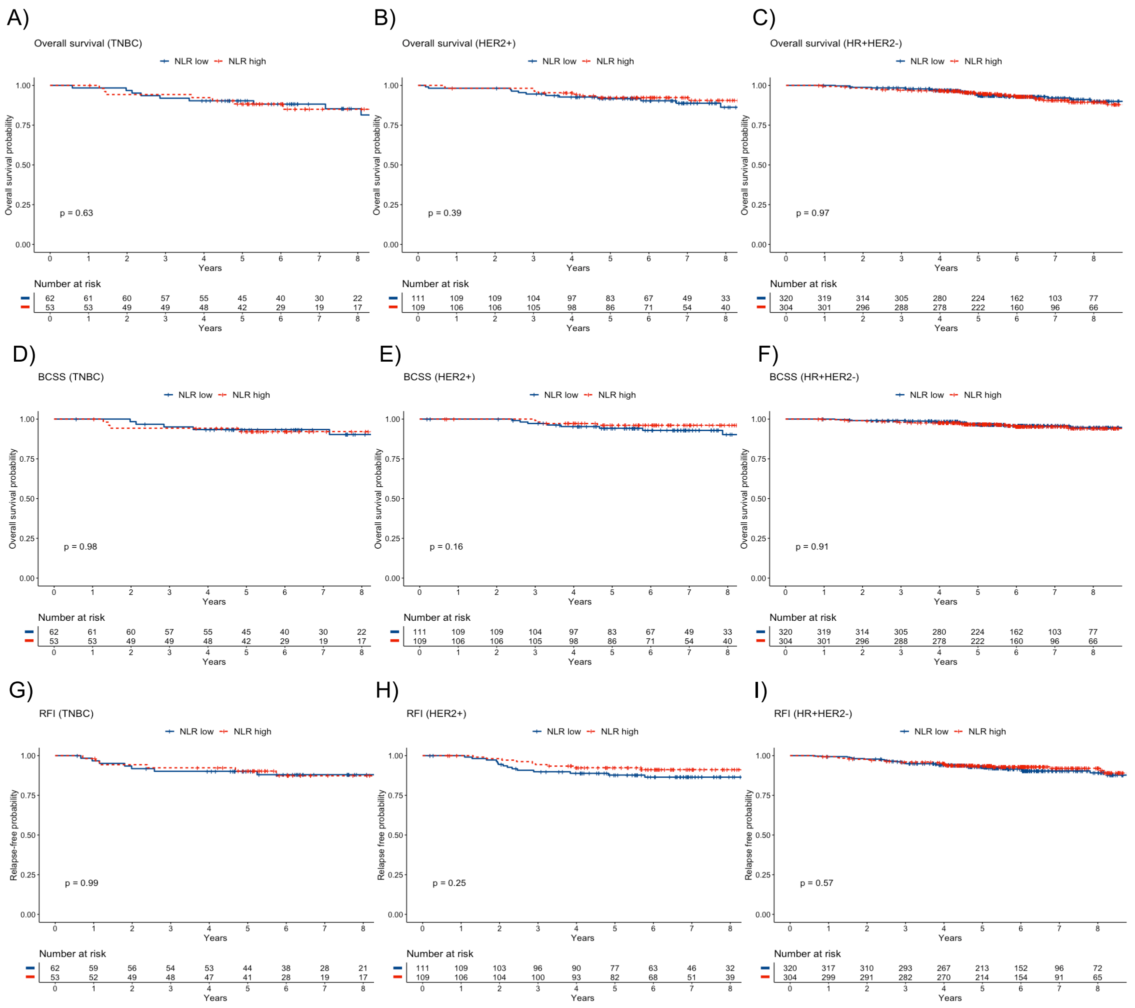

Fig. S7 Kaplan-Meier survival curves for baseline NLR across breast cancer subtypes (triple negative breast cancer, HER2+, HR+/HER2-) (A, B, C) Overall survival (OS); (D, E, F) Breast cancer specific survival (BCSS); (G, H, I) Relapse free interval (RFI). P-values correspond to log-rank test.
